# Predicting Market Impact Costs Using Nonparametric Machine Learning Models

**DOI:** 10.1371/journal.pone.0150243

**Published:** 2016-02-29

**Authors:** Saerom Park, Jaewook Lee, Youngdoo Son

**Affiliations:** 1 Department of Industrial Engineering, Seoul National University, Seoul, South Korea; 2 Research Institute of Engineering and Entrepreneurship, Seoul National University, Seoul, South Korea; 3 Department of Industrial and Systems Engineering, Rutgers University, Piscataway, New Jersey, United States of America; Northwestern Polytechnical University, CHINA

## Abstract

Market impact cost is the most significant portion of implicit transaction costs that can reduce the overall transaction cost, although it cannot be measured directly. In this paper, we employed the state-of-the-art nonparametric machine learning models: neural networks, Bayesian neural network, Gaussian process, and support vector regression, to predict market impact cost accurately and to provide the predictive model that is versatile in the number of variables. We collected a large amount of real single transaction data of US stock market from Bloomberg Terminal and generated three independent input variables. As a result, most nonparametric machine learning models outperformed a-state-of-the-art benchmark parametric model such as I-star model in four error measures. Although these models encounter certain difficulties in separating the permanent and temporary cost directly, nonparametric machine learning models can be good alternatives in reducing transaction costs by considerably improving in prediction performance.

## Introduction

Transaction cost is one of the important factors that affect the investment performance and is usually classified into two major categories: explicit costs and implicit costs. Explicit costs, also called direct costs, are transaction costs that can be explicitly stated and measured. These costs include commissions, transaction fees, and taxes. Implicit costs, or indirect costs, are costs that cannot be measured directly but can be improvable by an appropriate trading strategy. They include bid-ask spreads, time risk costs, and market impact costs.

Market impact cost, one of the implicit transaction costs, is the cost caused by the difference between the price before the transaction and the actual price that the transaction is executed actually. During the last decade, many studies have been focused on analyzing market impact costs by not only the academic researchers but also the practitioners because it is one of the main reducible parts of the transaction cost. [1] and [2] fitted the impacts of single transactions to a concave power-law function of the volume of the transaction. [3] used a logarithm function of the transaction volume to estimate market impact costs. [4] exploited the hyperbolic tangent function for the same task. [5] and [6] used a stochastic process of the asset price which includes a function of the transaction size to explain market impacts. [7] estimated impact cost by using a linear regression with quantized transaction sizes. [8] analyzed the market impacts of the large institutional orders in the US equity market and found that the permanent impact function has a concave form with respect to the transaction size, in contrast to the previous results [5, 9] in which the permanent impact function had a linear form. The I-star model, a state-of-the-art parametric model, described in [10] and [11] is a log-linear regression model that uses three inputs, transaction size, volatility, and underlying trading rate. These inputs affected the estimated market impact costs independently. [12] and [13] used more than 40 independent variables to fit the market impact cost to simple linear regression function. However, those existing parametric approaches showed the limitation in estimation and prediction performance because they assumed the fixed parametric or simple linear regression form of the market impact model. In addition, most of them cannot employ the variables that are not originally included in the model thus a new model is always required to predict the market impact with the different set of variables.

Recently, nonparametric machine learning models have been successfully applied to diverse financial applications because of their abilities in fitting and predicting performances for complex data sets. They include the stock price prediction [14–17] and its derivative markets [18–20], credit and its derivative markets [21–23], fixed-income markets [24, 25], and foreign exchange markets [26, 27].

In this paper, we introduce nonparametric machine learning approaches to estimate and predict market impact costs more accurately than the existing parametric approaches. To our knowledge, this is the first approach that applies several nonparametric learning models to analyze the market impact cost. The proposed nonparametric approach has two main advantages. First, the nonparametric approaches usually fit the data better than does the parametric case. Second, the nonparametric approaches are versatile in the number of input variables so the general procedure does not change, whereas the number or kinds of input variables change while the parametric approaches require the new parametric models in those cases. By simulation, we analyzed the market impact costs of transactions of small-cap, mid-cap, and large-cap stocks in US equity market both altogether and separately by selecting the same types of input variables with I-star model [10, 11] and compared the results.

The remainder of the paper is organized as follows. In the next section, we briefly explain the I-star model which is used as a parametric benchmark with three input variables. Then, we describe how to use nonparametric regression models to construct market impact cost functions. Data description and experimental procedures with the experimental results are presented in the following sections. Finally, we provide the summarized results, limitations, and some directions for the future work in the discussion section.

## Review of I-star model

In this section, we first briefly review I-star model [10, 11] which is a state-of-the-art benchmark parametric model. I-star model, which uses three input variables to describe the market impact cost, is composed of two separated equations calculating I*, a theoretical instantaneous cost, and *MI*, the market impact cost appeared in the real market, respectively. The equations calculate them are given as follows:

$$\begin{array}{cc} I^{*} & =a_{1}\cdot Size^{a_{2}}\cdot {Vol}^{a_{3}} \end{array}$$ (1)

$$\begin{array}{cc} MI & =b_{1}I^{*}\cdot POV^{a_{4}}+\left(1-b_{1}\right)I^{*} \end{array}$$ (2)

where *Size*, *Vol*, and *POV* are input variables and *a*_1_, *a*_2_, *a*_3_, *a*_4_, and *b*_1_ are parameters to be determined.

The first input variable of Eqs (1) and (2) is *Size*, the normalized order size. Based on [11], this variable is represented as *Size* = *Q*/*ADV*, where *Q* is the imbalance, the absolute value of difference between buy order and sell order, and *ADV* is 30-day average daily volume. Thus *Size* implies the magnitude of pressure from this order relative to the average daily volume. The second input variable, *Vol*, is the volatility of the equity return and 30-day averaged volatility was used in [11]. The last input variable, *POV*, is an acronym for *percentage of volume* and it reflects the market liquidity condition. [11] simply expressed *POV* = *Q*/(*Q*+*V*) where *V* is the expected volume traded for the period of time that the imbalance order *Q* is executed. If the market is liquid or the imbalance trade order *Q* is executed slowly, *V* becomes large and thus *POV* becomes small. Small *POV* results in small *MI* value so the market impact cost will be small when the market is liquid.

The market impact cost in Eq (2) comprises two components, temporary impact cost and permanent impact cost which are the first and the last term in the right hand side of Eq (2) respectively. Considering that *Size* and *Vol* are used to calculate the value of I*, they affect both the temporary and permanent part of the market impact. However, the other input variable *POV* only appears in the temporary impact part. This result implies that the smaller *POV* incurs the smaller market impact cost when the other input variables are invariant. However, this effect is temporarily and the permanent impact on the market is independent of the market liquidity condition.

Several parameters should be estimated. These parameters can be determined with data sets, including input variable values and market impact costs observed in the market, by general parameter estimation techniques such as nonlinear optimization and grid search.

## Nonparametric regression models

In this research, four state-of-the-art nonparametric machine learning models are used to estimate market impacts. As a preliminary, brief explanations of them are given as follows.

### Neural networks

Neural networks [28] are nonparametric nonlinear regression models which can be fit to highly nonlinear data distribution. Mimicking a human brain, a neural network model consists of layers that contains several nodes, conducting the same role with neurons in the human brain. Each node in the layer takes output values of all nodes in the previous layer as input values, calculates the output value, and provides the output value to all nodes in the next layer as one of their input values. The most common way of output value calculation in the node is as follows:

$$\begin{array}{c} y=f(\sum_{i}w_{i}g\left(x_{i}\right)) \end{array}$$ (3)

where *y* is the output value of the node, *x*_*i*_ represents input value from node *i* in the previous layer, *g*(⋅) is an input transform function, *w*_*i*_ represents weights for input values, and *f*(⋅) is an activation function that makes the regression model nonlinear. The sigmoid functions, such as logistic function, probit function, and hyperbolic tangent function, and a liner rectified function, for example, *f*(*x*) = max{0, *x*}, are usual selections for the activation function.

Finding optimal weights, *w*_*i*_, in Eq (3) is the main task of constructing the neural network model. The most extensively used method for this optimization *back-propagation algorithm* [29]. In back-propagation algorithm, the weights are modified, or the gradients are calculated, backward from the last output layer to the first input layer by minimizing the sum of squared errors as usual. Similar to other nonparametric regression methods, the neural network efectively finds the complex data distribution after optimizing weights. However, the relationship between input values and output values is difficult to determine.

### Bayesian neural networks

Bayesian neural network model, first proposed in [30], is a variant of the neural network model, whose weights have prior distribution similar to other Bayesian models. Maximizing the likelihood of this model is equivalent to minimizing the regularized error function, $E_{reg}\left(w,X,y\right)=E\left(X,y\right)+\lambda {∥w∥}_{k}^{k}$, where **w** is the weight vector, {**X**, **y**} are data inputs and outputs, *E*(⋅) is the error function, and ‖⋅‖_*k*_ is a k-norm function. If the prior distribution has a Laplace function or a Gaussian function, *k* has the value of 1 or 2, respectively. [31] proposed the iterative process of optimizing the Bayesian features including Bayesian neural networks by using Gauss-Newton approximation to compute the Hessian matrix of the objective error function *E*_*reg*_.

### Gaussian processes

Gaussian process regression [32, 33], a collection of random variables such that the distribution of any finite selection of them follows the joint Gaussian distribution, can be fully determined by the mean function and the covariance function which can be represented as follows:

$$\begin{array}{cc} m(x) & =E[f(x)] \end{array}$$ (4)

$$\begin{array}{cc} k(x,x^{\prime }) & =E[\left(f\left(x\right)-m\left(x\right)\right)\left(f{\left(x\right)}^{\prime }-m\left(x^{\prime }\right)\right)] \end{array}$$ (5)

where *f*(**x**) is the Gaussian process regression function and *m*(**x**) and *k*(**x**,**x**′) are its mean and covariance function respectively.

Suppose that the data set $D={\left\{\left(x_{i},y_{i}\right)\right\}}_{i=1}^{N}$ is given where the variance of the noise of the output values is denoted by *σ*^2^. Then, because the any finite joint distribution follows the multivariate Gaussian distribution described by Eqs (4) and (5), the likelihood of the Gaussian process model can be calculated as follows:

$$\begin{array}{c} \log P\left(y|D\right)=-\frac{1}{2}y^{T}{\left(K+\sigma^{2}I\right)}^{-1}y-\frac{1}{2}\log\det\left(K+\sigma^{2}I\right)-\frac{N}{2}\log2\pi \end{array}$$ (6)

where **y** = [*y*_1_, …, *y*_*N*_]^T^ and **K** is an *N*×*N* matrix whose ij’th component is *k*(**x**_*i*_,**x**_*j*_). Training Gaussian process means finding the hyperparameters in the mean and covariance functions and the output noise variance *σ*^2^; these values maximize the likelihood function in Eq (6). After finding those hyperparameters, the prediction for the new input x* can be estimated as

$$\begin{array}{c} \left[\begin{array}{c} y\\ f^{*} \end{array}\right]∼N\left(0,\left[\begin{array}{cc} K+\sigma^{2}I & k_{*}^{T}\\ k_{*} & k_{**} \end{array}\right]\right) \end{array}$$ (7)

where f* = *f*(x*), k_**_ = *k*(x*,x*), and k_*_ = (*k*(**x**_1_,x*), …, *k*(**x** *n*,x*))^*T*^. For more details for Gaussian processes, see [34].

### Support vector regression

Support vector regression proposed in [35] is a simple regression with a basis function *ϕ*(**x**) whose inner product can be represented a kernel function, <*ϕ*(**x**), *ϕ*(**x**′) > = k(**x**,**x**′) and an *ε*-insensitive loss function, $L\left(y_{1},y_{2}\right)=\max\{0,|y_{1}-y_{2}\left|-\varepsilon \right\}$, with some *ε* > 0. The value of this loss function is zero if |*y*_1_−*y*_2_|<*ε* thus it is called *ε*-insensitive.

Assuming the regression form as *f*(**x**, **w**) = < **w**, *ϕ*(**x**) > +*b*, the support vector regression problem is defined to minimize the sum of errors with the regularization which minimizes ∥**w**∥^2^ to reduce the complexity of the model as follows:

$$\begin{array}{c} \min_{w,b}\frac{1}{2}{∥w∥}^{2}+C\sum_{i=1}^{N}\left(\xi_{i}^{+}+\xi_{i}^{-}\right) \end{array}$$ (8)

with the constraints

$$\begin{array}{lll} y_{i}-f\left(x_{i},w\right) & \leq & \varepsilon +\xi_{i}^{+}\\ f\left(x_{i},w\right)-y_{i} & \leq & \varepsilon +\xi_{i}^{-}\\ \xi_{i}^{+},\xi_{i}^{-} & \geq & 0 \end{array}$$ (9)

for all *i* = 1, …, *N*. Applying Karush-Kuhn-Tucker conditions to the minimization problem above results in the following dual problem:

$$\begin{array}{c} \max_{\alpha^{+},\alpha^{-}}-\frac{1}{2}\sum_{i=1}^{N}\sum_{j=1}^{N}\left(\alpha_{i}^{+}-\alpha_{i}^{-}\right)\left(\alpha_{j}^{+}-\alpha_{j}^{-}\right)k\left(x_{i},x_{j}\right)-\varepsilon \sum_{i=1}^{N}\left(\alpha_{i}^{+}-\alpha_{i}^{-}\right)+\sum_{i=1}^{N}\left(\alpha_{i}^{+}-\alpha_{i}^{-}\right)y_{i} \end{array}$$ (10)

with the constraints $0\leq \alpha_{i}^{+},\alpha_{i}^{-}\leq C$ for all *i* = 1, …, *N*. Then, the solutions for the primal problem are becomes

$$\begin{array}{lll} w & = & \sum_{i=1}^{N}\left(\alpha_{i}^{+}-\alpha_{i}^{-}\right)\phi \left(x_{i}\right)\\ b & = & y_{k}-\sum_{i=1}^{N}\left(\alpha_{i}^{+}-\alpha_{i}^{-}\right)k\left(x_{i},x_{k}\right) \end{array}$$

for any *k* = 1, …, *N*. After solving the dual problem by using a quadratic programming solver, the predictive value for a new input x* becomes

$$\begin{array}{c} y^{*}=\sum_{i=1}^{n}\left(\alpha_{i}^{+}-\alpha_{i}^{-}\right)k\left(x_{i},x^{*}\right)+b \end{array}$$ (11)

which can be represented without the basis function *ϕ*(**x**) itself but only with its inner product kernel function *k*(**x**, **x**′).

## Data description and procedures

In this section, we describe the proposed procedure to calculate the market impact cost by using nonparametric machine learning models with an example of single transaction data of representative US stocks.

### General procedures

**Fig 1:**
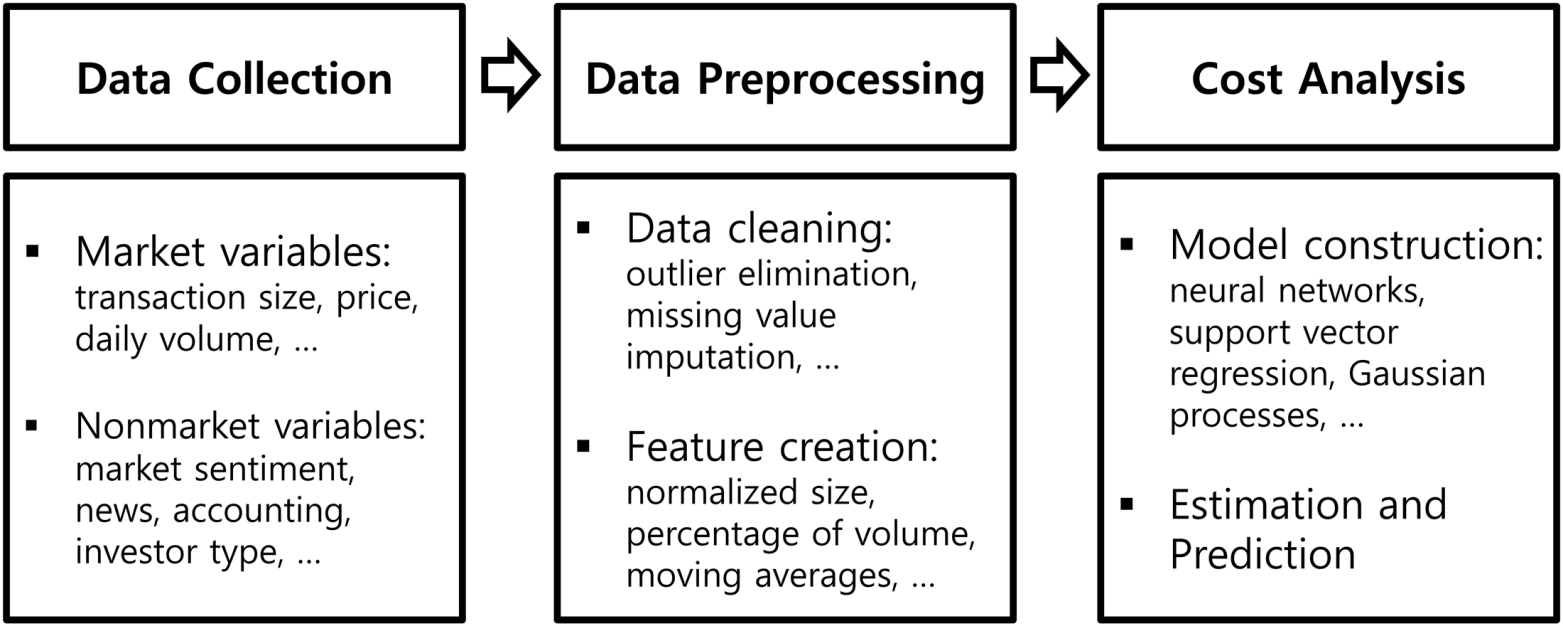
Summary of the general procedure of nonparametric approach for market impact cost.

First, we suggest the general procedure to find market impact costs by using nonparametric regression models before the descriptions of the simulation conducted in the current paper. The whole procedure is classified into three stages: data collection, data preprocessing, and cost analysis. Fig 1 represents the summary of the whole procedure.

The main task at the first stage is data collection, which aims to gather necessary data. Collecting non-traditional data outside the market such as news, reports, opinions, and any other variables than may affect price or liquidity can also be useful as well as the traditional market variables because the nonparametric models do not require any restriction on the data and the general procedure of analyzing market impact costs using them will not be changed.

The gathered data from the first stage are preprocessed to make input variables that are used for learning process in the second stage. First, data cleaning processes such as outlier elimination and missing value imputation are conducted. Then, the input variables that will be used for the nonparametric models are derived from these cleaned data.

At the final stage, nonparametric models to estimate and predict market impact costs are constructed using the input variables created in the previous stage. In addition, any other analyses using the constructed model including testing statistical significance can be conducted at this stage.

### Data description

For simulating the proposed nonparametric approach, we gathered a single transaction data of the stocks of US equity markets from Bloomberg Terminal for the period from 2014/06/02 to 2014/06/26. We selected 17 representative firms that have large market capitals among each of S&P 500, S&P MidCap 400, and S&Ps SmallCap 600 indices. These indices are composed of large cap, mid cap, and small cap firms respectively. The tickers of the selected firms are presented in Table 1.

**Table 1:** Tickers of selected firms.

*Large cap*	*Mid cap*	*Small cap*
AAPL	ADS	FNGN
XOM	AMG	TDY
GOOGL	GMCR	WST
GOOG	TSCO	DAR
MSFT	MHK	WWW
JNJ	LKQ	TYL
WFC	HFC	TTC
GE	HSIC	CGNX
CVX	DDD	QCOR
WMT	PII	CNC
JPM	UA	ENS
PG	CHD	MDSO
VZ	BEAV	LHO
IBM	XEC	VSAT
PFE	JBHT	MMS
T	TRMB	VDC
ORCL	EQIX	SF

A total of 17 firms with large market capitals among each of large, mid, and small cap indices by S&P are chosen.

The collected transactions data are classified into three data sets, *large cap*, *mid cap*, and *small cap* by their capitals; another data set *all cap* includes all transactions regardless of the market capital. For each size of capitals, the number of collected transactions are approximately 15 million, 2 million and 1 million for *large cap*, *mid cap*, and *small cap*, respectively. Thus the *all cap* data set has approxiamtely 18 million transactions in total. The procedures in the following sections will be applied commonly to all of those data sets.

### Creating and bucketing input variables

We made three key input variables, *Size*, *Vol*, and *POV*, which are also used in I-star model [10, 11] and one output variable, the market impact cost. Considering that the I-star was originally applied to the daily-aggregated transactions, we slightly modified the input variables suitable for single high-frequent transactions. First, we define the market impact cost, denoted by *cost*, as

$$\begin{array}{c} cost=side\cdot \log\left(p_{t}/p_{0}\right)\cdot 10^{4} \end{array}$$ (12)

where *side* is 1 if a trade is a buy-initiated trade and −1 if a trade is a sell-initiated trade, *p*_0_ is a mid-price just before the trade, and *p*_*t*_ is an executed price of the trade. Given that *cost* is multiplied by 10^4^, the unit of *cost* becomes basis point (bp). The first input variable *Size* is the normalized trade size as follows:

$$\begin{array}{c} Size=\frac{V_{t}}{ATV} \end{array}$$ (13)

where *ATV* is the average trade volume of the previous day. In the original I-star model, the imbalanced trade size is normalized by 30-day average daily volume because the trade size itself is daily = aggregated. In our research, to apply the single transactions, we divide each trade size by the one-day average of the single trades of the previous day. The second input variable *Vol* is defined as the 30-day volatility, and this value is the same with the original I-star model because volatility is the characteristic of each stock, unrelated to trade size or frequency. *POV*, the percentage of volume, in [11] is defined as *Q*/*V* where *Q* is the daily imbalanced size and *V* is the total trade volume of that day. A single transaction may be affected by market liquidity more locally rather than the liquidity of the whole day. Thus we define *POV* for single transactions as

$$\begin{array}{c} POV=\frac{V_{t}}{V_{t}\left(-\tau ,\tau \right)} \end{array}$$ (14)

where *V*_*t*_(−*τ*, *τ*) is the total traded volume from *τ* minutes before the trade to *τ* minutes after the trade. Based on the previous study [7], we expected that the single transaction affects and is affected by the market within approximately 15 minutes and thus we decided that *τ* equals 15.

After creating input variables, we made three dimensional bins of input variables and bucketed the transactions into them. For each bin, *Size* has values of multiples of 0.01, i.e. 0,0.01,0.02,…, and *Vol* has values of multiple of 0.05. *POV* has the values of multiples of 0.0002 for the *large cap* data set and multiples of 0.001 for the other types of data sets. Each transaction was bucketed to the bin with the nearest value. For example, a transaction from *mid cap* data set with the input variables (*Size*, *Vol*, *POV*) = (0.0137,0.022,0.0038) was put to the bin with the values (*Size*, *Vol*, *POV*) = (0.01,0.02,0.004). The output, *cost*, of each bin is defined by the average *cost* of transactions belonging to the bin.

Finally, we selected bins containing enough number of transactions. The criterion number will be different for data sets. We selected the bins containing more than 20, 30, 60, 100 transactions; the number of survived bins are 2931, 3356, 5706, 5119 for *small cap*, *mid cap*, *large cap*, and *all cap*, respectively.

### Analyzing market impact costs

With respect to the bins of transactions to be used for the nonparametric machine learning models, we set 70% of survived bins as the training set and the remaining 30% as the test set for each data set. To find appropriate parameter sets of nonparametric models, we used 10-fold cross validation for the training set. After finding the parameter set, each model was retrained for the entire training set with the chosen parameter set and applied to the test set. As a parametric benchmark, we used I-star model with the same data sets. As described in Section, I-star model also requires finding certain parameters. We found the parameters for I-star model by grid search and 10-fold cross validation of the training set and applied it to the test set as the same with the nonparametric models. Finally, we applied Wilcoxon signed-rank test between each nonparametric model and the parametric benchmark for each capital size group to find whether the difference in performance between a nonparametric model and the benchmark is significant.

## Results

### Predicting market impact costs

First, we applied the nonparametric machine learning models and the benchmark parametric model, I-star model, to the selected bins of each data set. To estimate the errors of the model, we used four different measures, mean absolute error (MAE), relative MAE (RMAE), root mean squared error (RMS), and relative RMS (RRMS). The summarized results are shown in Tables 2–5. *NN*, *BNN*, *SVR*, *GP*, and *I-star* refer to neural network, Bayesian neural network, support vector regression, Gaussian process, and I-star model, respectively.

**Table 2:** Test errors of the nonparametric models and the parametric benchmark models for *small cap* data set.

Methods	MAE	RMAE	RMS	RRMS
*NN*	0.9445 (0.9910)	0.3006 (0.3175)	1.4945 (1.5305)	0.4535 (0.4990)
*BNN*	0.9310 (1.0025)	0.3023 (0.3204)	**1.4559** (1.5286)	0.4502 (0.4820)
*GP*	**0.8794** (0.8701)	**0.2854** (0.2716)	1.4945 (1.3950)	**0.4442** (0.4060)
*SVR*	1.0121 (1.0333)	0.3352 (0.3373)	1.5783 (1.5762)	0.5090 (0.5340)
*I-star*	1.0396 (1.0446)	0.3410 (0.3408)	1.5701 (1.5891)	0.5097 (0.5476)

Cross validation errors are also displayed in the parentheses. The best model for each error measure is **boldfaced**.

**Table 3:** Test errors of the nonparametric models and the parametric benchmark models for *mid cap* data set.

Methods	MAE	RMAE	RMS	RRMS
*NN*	**0.5266** (0.5254)	0.2831 (0.2932)	**0.7851** (0.7542)	0.4184 (0.4381)
*BNN*	0.5405 (0.5186)	0.2914 (0.2889)	0.7892 (0.7423)	0.4188 (0.4338)
*GP*	0.5517 (0.5178)	**0.2802** (0.2778)	0.8311 (0.7597)	**0.3907** (0.4144)
*SVR*	0.6202 (0.3251)	0.3268 (0.3251)	0.8914 (0.8358)	0.4672 (0.4746)
*I-star*	0.6540 (0.6226)	0.3453 (0.3424)	0.9373 (0.8730)	0.4972 (0.5080)

Cross validation errors are also displayed in the parentheses. The best model for each error measure is **boldfaced**.

**Table 4:** Test errors of the nonparametric models and the parametric benchmark models for *large cap* data set.

Methods	MAE	RMAE	RMS	RRMS
*NN*	0.1287 (0.1283)	0.1515 (0.1506)	0.1732 (0.1738)	**0.2051** (0.2054)
*BNN*	**0.1267** (0.1280)	**0.1502** (0.1505)	**0.1712** (0.1735)	0.2066 (0.2061)
*GP*	0.1338 (0.1377)	0.1583 (0.1621)	0.1802 (0.1878)	0.2172 (0.2123)
*SVR*	0.1872 (0.1896)	0.2267 (0.2300)	0.2466 (0.2459)	0.3085 (0.3112)
*I-star*	0.2203 (0.2229)	0.2635 (0.2661)	0.2823 (0.2823)	0.3484 (0.3503)

Cross validation errors are also displayed in the parentheses. The best model for each error measure is **boldfaced**.

**Table 5:** Test errors of the nonparametric models and the parametric benchmark models for *all cap* data set.

Methods	MAE	RMAE	RMS	RRMS
*NN*	0.4096 (0.3746)	0.4096 (0.2173)	0.7557 (0.6388)	0.3507 (0.3210)
*BNN*	**0.3789** (0.3683)	**0.2182** (0.2170)	**0.6667** (0.6251)	**0.3192** (0.3292)
*GP*	0.4327 (0.4059)	0.2586 (0.2519)	0.7383 (0.6598)	0.3601 (0.3576)
*SVR*	0.4488 (0.4256)	0.2766 (0.2710)	0.7485 (0.6840)	0.3933 (0.3964)
*I-star*	0.4747 (0.4517)	0.2989 (0.2931)	0.7784 (0.7029)	0.4163 (0.4149)

Cross validation errors are also displayed in the parentheses. The best model for each error measure is **boldfaced**.

**Fig 2:**
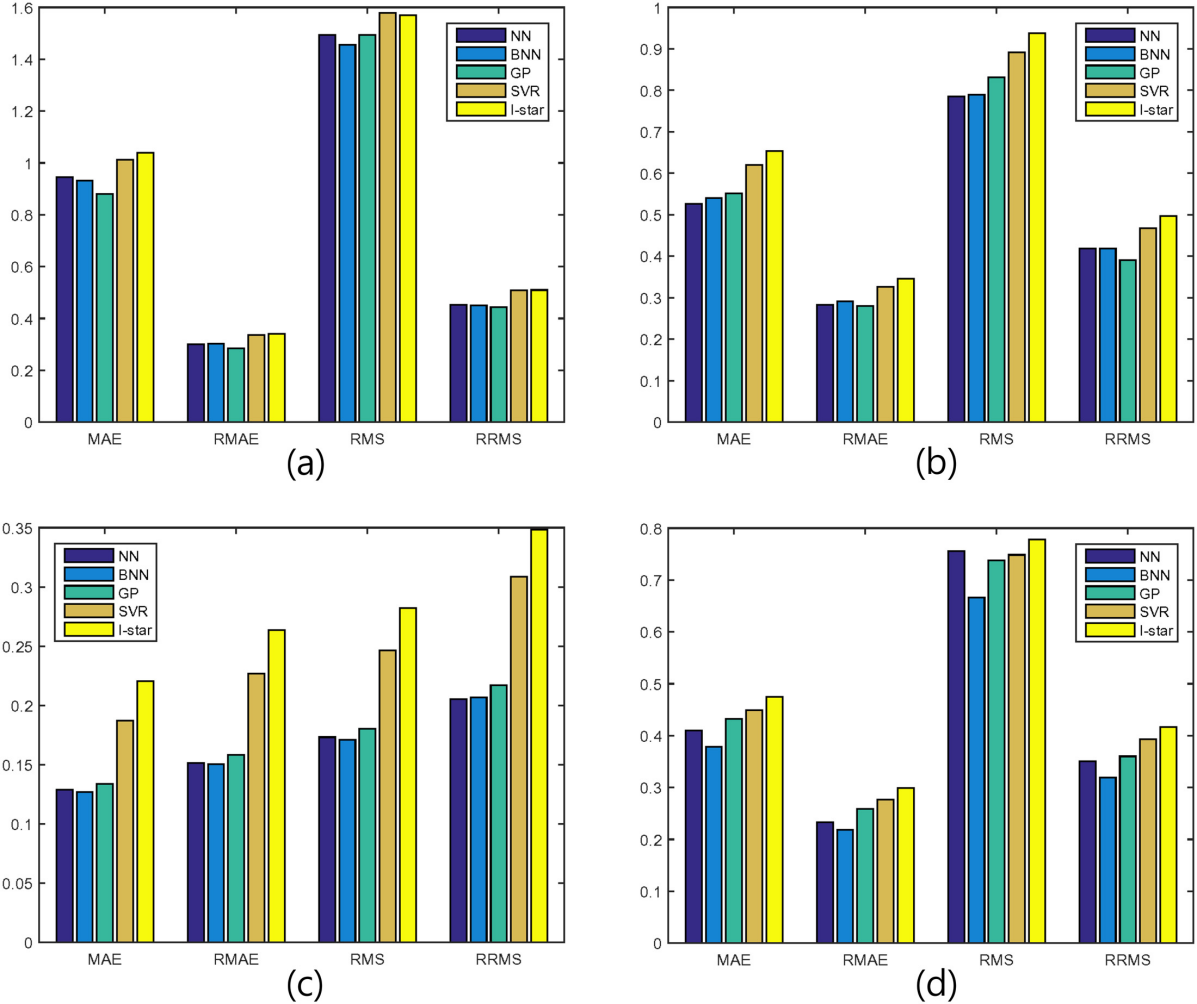
Test errors of the nonparametric machine learning models and the parametric benchmark. (a) *small cap* data set (b) *mid cap* data set (c) *large cap* data set (d) *all cap* data set.

From Tables 2–5, all the nonparametric machine learning approaches fit the data distribution better than does the parametric benchmark with the same input features and instances, as expected. Secondly, the compared nonparametric machine learning models indicated different performances. For example, Bayesian neural networks reduced the errors from 7.27% to 43.00% relative to I-star model but support vector regression reduced the errors just from -0.005% to 15.03%. This phenomenon is more clarified by Fig 2 which represented the errors in the tables above.

In summary, we find that the three nonparametric models, neural network, Bayesian neural network, and Gaussian process, shows much better performances than the parametric benchmark while support vector regression model performs slightly better than the benchmark and worse than the other nonparametric models in general. In some measures such as RMS, support vector regression performs slightly worse than the benchmark model for *small cap* data set.

To validate the proposed approach, in addition, we applied the Wilcoxon signed-rank test to each pair of the instance-wise test errors of one nonparametric model and the benchmark parametric model for each error measure. The p-values obtained by normal approximation of the Wilcoxon signed-rank test and all of them were smaller than 0.05, which is usually considered as a critical value of the statistical significance. Even though the averaged RMS of SVR prediction is larger than the benchmark, the predicted performance of SVR was judged significantly better than the parametric benchmark by the Wilcoxon signed-rank test.

## Discussion

In this study, we introduced the nonparametric approaches to estimate and predict market impact costs and applied them to US stock markets with the three input variables used in I-star model, the parametric benchmark. This study has several features. First, four state-of-the-art nonparametric machine learning models including NN, BNN, GP, and SVR have been applied to the task of analyzing market impact cost, whereas the previous studies were focused on only parametric models. The nonparametric machine learning approaches have advantages in both prediction performance and versatility in the number of input variables. Second, the data set used in this study was highly extensive. A total 17 firms were selected from each indices of large, mid, and small cap firms, while the previous studies mostly focused on the large cap firms. The total amount of transactions used in this study exceeded 18 million. Finally, the market impact prediction in this paper used independent variables from single transactions. Thus, this prediction has the advantages to be applied to technological high-frequency trades compared with previous studies that analyzed only large trades.

As a result of the experiments performed in this study, the performances of nonparametric machine learning models mostly overwhelmed the benchmark model with the same input variables for all kinds of firm sizes and all error measures. In particular, BNN, NN, and GP showed noticeably better performances, whereas SVR sometimes performed worse than or comparably to the benchmark model. The statistical significance of the predictive powers of nonparametric approaches was also validated by applying the Wilcoxon signed rank test to the test error.

However, one of the limitations of the nonparametric machine learning approaches is that they cannot directly separate the market impact cost into permanent and temporary cost, whereas some parametric models can. Considering that the total, or instantaneous impact primarily affects the price at which the transaction occurs, an indirect way to analyze the permanent or temporary portion of it using nonparametric models can be helpful in analyzing market characteristics. In addition, though the nonparametric models usually performed better than did the parametric benchmark with the same input variables, the magnitude of performance difference can be changed if the period or the selected firms vary.

This study implies possibilities to be extended on some points. First, the nonparametric machine learning models has the advantage over parametric models in that the input variables can be added freely without any limitations. Thus, studies related to the nonmarket variables affecting the market impact can be easily incorporated into nonparametric models rather than parametric ones which are formed with a priori fixed input variables. Next, several parametric models explain the market impact cost. However, they are difficult to compare because their input variables are varied, as is their number. In such cases, a nonparametric machine learning model with the same inputs as the parametric models can provide a performance benchmark. Finally, developing hybrid models of nonparametric and parametric ones that comprise the permanent and temporary portions of the market impact cost as well as that maintain the extendability and the performance level remains a future research topic related to this study.
